# Efficacy of nonsurgical tigecycline pleurodesis for the management of hepatic hydrothorax in patients with liver cirrhosis

**DOI:** 10.1186/s40792-015-0049-x

**Published:** 2015-08-12

**Authors:** Nevin Yilmaz, Arife Zeybek, Benjamin Tharian, Ugur Eser Yilmaz

**Affiliations:** Transplant Hepatology, Mugla University School of Medicine, 48000 Mugla, Turkey; Thoracic Surgery, Mugla University School of Medicine, Mugla, Turkey; Department of Medicine and Gastroenterology, NWAHS, Tasmania, Australia; Royal College of Surgeons in Ireland Medicine School (RCSI) / Graduate Entry Program, Dublin, Ireland

## Abstract

Chemical pleurodesis is one of the therapeutic tools to control hepatic hydrothorax. Tetracycline and derivatives have been widely accepted as an effective and safe treatment for the purpose, but availability is the big concern. Tigecycline is an antibiotic derivative of tetracycline, which has demonstrated to be an effective pleurodesing agent in animal models. The aim of the study was to document two successful tigecycline pleurodesis in patients with decompensated liver cirrhosis, who were not candidates for liver transplantation. Both patients were undergoing palliative treatment for cirrhosis and developed massive pleural effusion on the right side. They underwent chemical pleurodesis in the first instance. Diagnostic thoracocentesis was done to rule out differentials and to confirm the clinical suspicion, following which, complete drainage of pleural fluids was achieved. Tigecycline of 3 mg/kg was instilled intrapleurally via the thoracic catheter, as per the protocol. The medical records and images were thoroughly reviewed. There was no recurrence of the effusion for at least 3 months, with no detected complications in the short- or long-term follow-up. In conclusion, pleurodesis with tigecycline seems to be effective and safe for the management of symptomatic hepatic hydrothorax and should therefore be promoted in the setting of liver cirrhosis at least for a short-term relief, especially in patients who do not meet the criteria for liver transplantation.

## Background

Pleural effusion is a relatively uncommon complication of end-stage liver disease, which occurs in less than 10 % of the patients, typically located on the right side in approximately 70–80 % of the cases. Although right-sided pleural effusion can develop in the absence of significant ascites, coexisting ascites is normally present. The negative intrathoracic pressure draws ascitic fluid into the pleural space via the multiple natural defects in the diaphragm, like the “Pores of Kohn” [1–6].

Fluid analysis and complications will show characteristics similar to abdominal ascites. Although the treatment of hepatic hydrothorax (HH) is similar to the treatment of ascites, more invasive procedures including thoracocentesis that rarely have undesirable outcomes are sometimes needed. In refractory patients with HH, repetitive thoracocentesis can be performed in selected patients but always carries risk for pneumothorax and contamination [7–9].

The patient with recurrent or large-volume hydrothorax who is unresponsive to diuretics, transjugular intrahepatic portosystemic shunt (TIPS) can serve as a bridge to liver transplantation. Surgical closure of diaphragmatic defects has been also proposed, but the increased morbidity and mortality of these complex procedures require careful consideration of risk versus benefit [10–15].

Chemical pleurodesis with chest drainage is another treatment option to be strongly considered to give a good palliation to these patients. As is already known, the irritants cause reaction between the parietal and visceral layers of the pleura, which closes off the space between them and prevents further fluid from accumulating. On the other hand, the technique can sometimes be challenging to achieve pleurodesis in HH because of the rapid fluid accumulation [16, 17].

To date, a large number of chemicals were considered to induce pleurodesis in malignant or non-malignant effusions. Although talc, tetracycline derivatives, OK-432, bleomycin, or povidone–iodine are the most commonly used agents for liver-related pleurodesis, availability, side effects, and variable results are still major concerns [16, 18–25].

There are ongoing animal studies with different sclerosing agents. Tigecycline is the first drug in the glycylcycline class (group 3) of tetracyclines that is available for intravenous infusion with broader spectrum cover. The structure of the drug is very similar to minocycline and similarly binds to the bacterial 30S ribosome unit. The main difference between tigecycline and minocycline is the addition of an *N*,*N*-dimethylglycylamido group. Given the molecular differences, tetracycline galactosidase (TG) has pharmacokinetic distinction consistent with increased distribution and concentration in human tissue beyond the plasma volume. This indicates tigecycline penetrates with higher concentration in the pleural surfaces. Daddi and friends reported effectiveness of tigecycline pleurodesis in animal models, recently [26–28]. However, there is no published human study with tigecycline as a pleurodesing agent that we could find during our literature search. Our institute has historically used tetracycline derivatives to induce pleurodesis. We aim to demonstrate the safety and effectiveness of tigecycline pleurodesis, in patients with cirrhosis who are not transplant candidates.

## Case presentation

### Case 1

A 63-year-old man with cirrhosis secondary to hepatitis B infection was admitted to the hospital, based on the abnormal chest X-ray performed by gastroenterologist on his regular follow-up visits.

He had chronic symptoms of underlying liver disease, fatigue, abdominal swelling with ankle edema, cough, and exertional dyspnea. On further questioning, he reported worsening dyspnea over the past 5 to 6 weeks.

His past medical history was significant for bilateral inguinal hernia operation and COPD related to smoking. The liver disease was diagnosed in 2003, complicated with portal hypertension (esophageal varices (previously banded) and gastric varices) and ascites, which needed frequent paracentesis. He is on entecavir since 2010 and seroconverted but withdrawn from the transplant list due to portal vein thrombosis (PVT), which he developed recently. The other medications included furosemide, spironolactone, propranolol, proton-pump inhibitor, Osmolac, inhaled beta agonist (formoterol), and anticholinergics (ipratropium). Large-volume paracentesis with albumin infusion was performed as and when needed.

On physical examination, vital signs were T 37 °C, BP 120/70 mmHg, P 90/min, RR 25 cycles/min, and O_2_ sat 95 % in room air. General appearance was remarkable with pallor and mild respiratory distress. Abnormal findings on physical examination were bilateral pitting ankle edema, spider nevi, palmar erythema, jugular venous distension with one-sixth systolic murmur at apex, gynecomastia, decreased breath sounds along with dullness to percussion on two thirds of the right lung, and mild to moderate ascites with no flapping tremor.

The initial work-up was as follows: CBC, PT/APTT, LFT, electrolytes, and CXR. The other tests including fluid analyses are summarized in the tables. Other imaging including abdominal ultrasound, Doppler study, CT chest, and echocardiography were also obtained as part of the work-up of the right-sided effusion (Tables 1 and 2).

**Table 1:** Laboratory results of the patients

	Case 1			Case 2		
	Before pleurodesis	After pleurodesis, first 2 weeks (median)	Late follow-up	Before pleurodesis	After pleurodesis, first 2 weeks (median)	Late follow-up
CBC						
WBC, 10^3^/mm^3^	3	1.6^a^	4.4	5.9	7.1	4.4
Hb, g/dl	10.3	8.6^a^	11.3	10	10.6	9.4
Plt, 10^3^/mm^3^	49	32	34	150	158	114
INR, range (0.8–1.2)	1.49	2.2^a^	1.6	1.14	1.19	1.17
AST/ALT, IU/l	59/47	50/30	68/77	36/127	23/17	26/13
GGT/AP, IU/l	17/97	12/71	47/81	26/81	37/127	36/85
T.bil/D.bil, mg/dl	2.1/0.63	2.2/ 0.4	3.74/1.07	1.08/0.3	1.1/0.2	1.4/0.39
Albumin, g/dl	2	2.3	2.4	2.6	2.7	3
Creatinine, mg/dl	0.6	0.57	0.6	0.7	0.7	0.7
Na, mEq/l	134	133	132	141	133	140
MELD score	14	18^a^	17	9	9	9
CTP class	B	B	B	A	A	A

Abbreviations: *CBC* complete blood count, *INR* international normalized ratio, *ALT* alanine aminotransferase, *AST* aspartate aminotransferase, *GGT* gamma-glutamyl transpeptidase, *AP* alkaline phosphatase, *Na* sodium, *K* potassium, *CTP* Child–Turcotte–Pugh, *MELD* model for end-stage liver disease, *Plt* platelet, *T.bil* total bilirubin, *D.bil* direct bilirubin

^a^Pathologic changes

**Table 2:** Pleural fluid and diagnostic studies

Findings	Case 1	Case 2
Pleural fluid		
WBC, 10^3^/mm^3^	150	100
Pleural/serum protein (ratio)	<0.5	<0.5
SPAG	>1	>1
Pleural LDH, IU/l	NA	49
Culture	Negative	Negative
Cytology	Negative	Negative
Tigecycline doses, total (mg)	150	200
Pleural drainage		
Before the PL, ml	2050	3500
Following PL, ml	600	500
Duration of drainage, days	10	14
Abdominal ultrasonography	Right massive pleural effusion with atelectasis left kidney stone (6 mm); liver: contour nodular, coarsened echo texture, C/RL >7, SM; ascites	Bilateral pleural effusion (right > left); liver: irregular external contour SM; minimal ascites
Doppler ultrasound following pleurodesis	Liver: contour nodular, coarsened echo texture SM, collaterals in the perisplenic region, portal venous thrombosis	Liver, coarsened echo texture, SM, patent vascular structure
Echocardiography	EF 55 %, 1° TR, 1° MR, left ventricular hypertrophy	EF 65 %, 1° TR, left atrial dilatation, aortic valve, and mitral annular calcification

Abbreviations: *LDH* lactate dehydrogenase, *SPAG* serum–pleural fluid albumin gradient, *EF* ejection fraction, *TR* tricuspid regurgitation, *MR* mitral regurgitation, *C*/*RL* caudate–right lobe ratio, *PL* pleurodesis, *NA* not available

### Case 2

A 76-year-old female was brought to the emergency room with a history of worsening dyspnea. She was found to have massive right-sided pleural effusion and hence was admitted to thoracic surgery ward.

Past medical history was remarkable for umbilical hernia repair in the 1980s, large hiatal hernia with Cameron ulcers, rheumatoid arthritis, and COPD. A kidney stone and thyroid nodule were diagnosed during the current admission. Cryptogenic cirrhosis was diagnosed in 2009 complicated with portal hypertension (esophageal varices banded once) and minimal ascites (never tapped) but underwent pleural drainages secondary to HH twice in the past 2 months. She has not been listed for transplant because of the age and low model for end-stage liver disease (MELD) score. She was on Ursofalk, spironolactone, propranolol, and proton-pump inhibitor. Intravenous acetyl cysteine, albuterol, and budesonide nebules with continuous low-dose oxygen inhalation were added in the current hospitalization.

On physical examination, vital signs were T 37.2 °C, BP 130/90 mmHg, P 72/min, and O_2_ sat 86 % in room air. General appearance was noticeable with pallor, respiratory distress improving on sitting position, and cough on exertion. Abnormal findings were spider nevi, jugular venous dilatation, and decreased breath sounds with dullness to percussion on the right side. Swan neck deformities of the digits, incisional hernia, and doubtful ascites were the other accompanied findings.

The diagnostic tests and images are summarized below (Tables 1 and 2). Schirmer’s test (4 mm/5 mm in 5 min) and rheumatoid factor were also positive.

#### Tigecycline pleurodesis

**Fig. 1:**
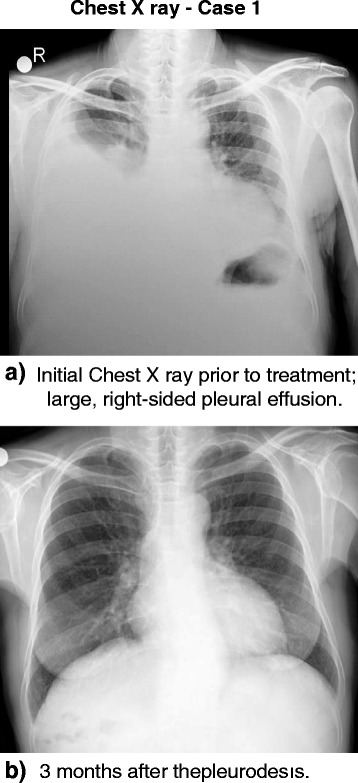
Chest X ray—case 1. **a** Initial chest X ray prior to treatment: large, right-sided pleural effusion. **b** Three months after the pleurodesis

**Fig. 2:**
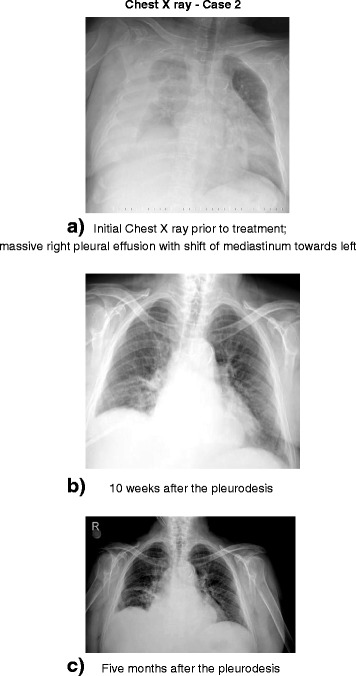
Chest X ray—case 2. **a** Initial chest X ray prior to treatment: massive right pleural effusion with shift of mediastinum towards left. **b** Ten weeks after the pleurodesis. **c** Five months after the pleurodesis

Imaging studies confirmed right-sided free-flowing pleural effusion and the aspirated fluid characteristic of transudate in both patients (Figs. 1a and 2a, Table 2). Patients were consented for chest drain insertion and pleurodesis with tigecycline as per institution protocol, after getting ethical committee approval. Ultrasound-guided therapeutic thoracocentesis was performed with a posterior intercostal approach with the patient seated, after the tissue at the entry site was anesthetized, a small incision was made, and an 18-gauge needle was directed into the pleural space. The needle was exchanged over the wire and 12-F tubes were connected to a drainage bag via a stopcock to perform gravity drain. The tube was intermittently clamped for 2 h to interrupt free drainage, to reduce the risk of re-expansion pulmonary edema. A total of 2050 ml from case 1 and 3500 ml from case 2 were obtained at the end of 3 days. Once the drainage dropped to <100 ml in a 24-h period and re-expansion of the lung was documented by chest X-ray, 3 mg/kg of tigecycline (diluted with 100 ml of saline) was instilled into the pleural space. Following the pleurodesis, the tube was clamped for 6 h and then unclamped. Once outputs were below 100 ml daily, they were clamped again.

The tubes remained 10 and 14 days in the patients with a total quantity of 500 and 600 ml fluid drainage, respectively. Meanwhile, the lungs had completely re-expanded and pleurodesis did not have to be repeated in the cases.

During the procedures and following days, the patients received prophylactic antibiotic therapy (intravenous ceftriaxone). Albumin infusion and diuretic doses were also tailored as was appropriate. None of the patients had experienced compensatory ascites accumulation following the procedures.

#### Follow-up

The patients experienced symptomatic improvement initially, and the procedures were tolerated without giving pain medication. During the following first 3 weeks, case 1 experienced drop in WBC–Hb and rise in international normalized ratio (INR) levels, temporarily (Table 1). During the initial few weeks, the median WBC was 1.6 10^3^/mm^3^ (range 1.31 to 2.7 10^3^/mm^3^) and Hb was 8.6 mg/dl (range 8.6–9.8 mg/dl). The patient needed 1 unit red blood cell transfusion. The median INR was 2.2 (range 1.6 to 2.2) and MELD score was 18. During the following 3 months, none of the patients experienced recurrence of effusions (Figs. 1b and 2b, c).

## Discussion

Hepatic hydrothorax is defined as a transudative pleural effusion, usually greater than 500 ml in patients with portal hypertension, without any other underlying primary lung diseases or alternative explanation for the same. Bilateral effusion develops in only 2–3 % patients and 20 % occurs without significant ascites. The pathogenesis of pleural fluid becomes complex in these patients. Therefore, some difficulties to establish therapeutic guidelines persist. Twenty percent of patients with HH are refractory to medical treatment and warrant consideration of invasive procedures as summarized before. Liver transplantation is the only one radical treatment, but most of the patients are not eligible for transplant like the cases discussed in the article or die while on waiting list. Consequently, thoracocentesis with or without pleurodesis, repair of diaphragmatic defects, and TIPS procedures have been proposed in the literatures [1–5].

In the presentation, both patients had massive pleural effusion on the right side. Case 1 had ascites with bilateral minimal pleural fluid reported previously in the sonographic examination which was not apparent in the chest X-ray. The other case had recurrent large fluid accumulations at the same location in a short time period (<2 months) without history of ascites. Like the first patient, she also had bilateral minimal effusion, only on ultrasonography. Neither had evidence of major defects in the diaphragms confirmed by thorax CTs. The clinical conditions showed they will not tolerate either repeated thoracentesis or any surgical procedures. We planned pleurodesis following diagnostic paracentesis and consented for the same.

Tigecycline is structurally similar to tetracycline class of antibiotics but has activity against many pathogens resistant to tetracyclines. Due to the special molecular structure, tigecycline has a highest steady-state volume of distribution (7 to 9 l/kg), higher in vitro plasma protein binding capacity (ranges from 71 to 79 %), and longest half-life with 36 h among the tetracyclines [27]. These characteristics indicate that tigecycline binds more tightly with higher concentration in the pleural surfaces with result of prolonged inflammation duration and higher sclerosant rate. Side effects are also similar to tetracyclines including photosensitivity, pseudotumor cerebri, and anti-anabolic action (which have led to increased BUN, acidosis, and hyperphosphatemia). As with tetracyclines, pancreatitis has been reported with the use of tigecycline. The recommended dose for the drug as an antibiotic is an initial dose of 100 mg followed by 50 mg every 12 h and the duration guided by the severity and the site of the infection. Fifty-nine percent of drug is eliminated by biliary/fecal excretion and 33 % in urine. No significant difference following tigecycline exposure was observed between healthy elderly subjects and youth, following a single 100-mg dose of tigecycline. But in patients with severe hepatic impairment (Child–Pugh C), dosage adjustment is arranted [26]. In the animal study, pleurodesis effect was apparent from 3 mg/kg and was dose dependent [28].

Both patients were older with mild to moderate hepatic impairment. Therefore, 3 mg/kg tigecycline was preferred. The procedures were well tolerated and metabolic tests remained within normal limits. Only case 1 with moderate (Child–Pugh B) liver cirrhosis experienced decrease in WBC below the 2 10^3^/mm^3^, and the prolonged INR (>2) was reversible after 2–3 weeks of treatment. In animal models, reversible bone marrow hypocellularity was recorded with tigecycline at exposures of 8–10 times the human dose. In clinical trials, anemia was observed in 5 % of patients [26]. Therefore, it is difficult to initiate the tigecycline therapy and bone marrow toxicity with a single-dose adjustment.

Singh and friends compared the management of HH and their results in the review [22]. Advanced thoracic procedures were used in almost all subjects, and follow-up durations were longer than our cases. Only two studies had follow-up limited to 3 months, but overall success rates were 75 and 67 % [15, 24]. In those studies, medical thoracoscopy and video-assisted thoracoscopic surgery (VATS) were used for pleurodesis and radiographic disappearance of fluid was defined as a success [15, 24]. In the first study, talc, vibramycin, and povidone–iodine were used as chemical agents and applied via medical thoracoscopy. Similar to the first case, all patients were in CTP class B. Differently, somatostatin was infused and pleurodesis was repeated at least once in all patients. The mean duration of drainage was 9.8 days (range 4–17 days), recurrence rate was 20 %, and complications range was 4 to 20 % [15].

In the second 3-month study, talc was applied using VATS or VATS with mechanical abrasion [24]. The recurrence rate was 25 % and the patients were re-treated with tetracycline. The maximum duration of post-pleurodesis drainage was longer (range 5–29 days) than our cases.

These results suggest that tigecycline pleurodesis was successfully achieved in these cases and they remained asymptomatic and hydrothorax free for 3 months follow-up. Only case 1 had temporarily hematologic and coagulation abnormalities. One of the disadvantages of the drug is a high commercial price. But compared to talc, easy biodegradability of the molecule makes the tigecycline available for using several times in the same patients with safety [27, 28]. The extent of talc systemically absorbed after intrapleural administration has not been adequately studied, and mutagenicity is not clear [29]. Among the tetracyclines, TG is the only one with IV form nationwide [27]. Therefore, tigecycline should be selected fairly as a pleurodesis agent based on the balance of clinical effect and cost benefits.

## Conclusions

In conclusion, pleurodesis with tigecycline is an effective and safe procedure in patients with hepatic hydrothorax. Long-term studies are necessary to define the optimal therapeutic guidelines, effectiveness, and side effects.

## Consent

The signed approval (informed consent) of the patients was received before the procedure.
